# Membrane Fusion and Cell Entry of XMRV Are pH-Independent and Modulated by the Envelope Glycoprotein's Cytoplasmic Tail

**DOI:** 10.1371/journal.pone.0033734

**Published:** 2012-03-27

**Authors:** Marceline Côté, Yi-Min Zheng, Shan-Lu Liu

**Affiliations:** 1 Department of Molecular Microbiology and Immunology, Christopher Bond Life Sciences Center, University of Missouri, Columbia, Missouri, United States of America; 2 Department of Microbiology and Immunology, McGill University, Montreal, Quebec, Canada; INSERM, France

## Abstract

Xenotropic murine leukemia virus-related virus (XMRV) is a gammaretrovirus that was originally identified from human prostate cancer patients and subsequently linked to chronic fatigue syndrome. Recent studies showed that XMRV is a recombinant mouse retrovirus; hence, its association with human diseases has become questionable. Here, we demonstrated that XMRV envelope (Env)-mediated pseudoviral infection is not blocked by lysosomotropic agents and cellular protease inhibitors, suggesting that XMRV entry is not pH-dependent. The full length XMRV Env was unable to induce syncytia formation and cell-cell fusion, even in cells overexpressing the viral receptor, XPR1. However, truncation of the C-terminal 21 or 33 amino acid residues in the cytoplasmic tail (CT) of XMRV Env induced substantial membrane fusion, not only in the permissive 293 cells but also in the nonpermissive CHO cells that lack a functional XPR1 receptor. The increased fusion activities of these truncations correlated with their enhanced SU shedding into culture media, suggesting conformational changes in the ectodomain of XMRV Env. Noticeably, further truncation of the CT of XMRV Env proximal to the membrane-spanning domain severely impaired the Env fusogenicity, as well as dramatically decreased the Env incorporations into MoMLV oncoretroviral and HIV-1 lentiviral vectors resulting in greatly reduced viral transductions. Collectively, our studies reveal that XMRV entry does not require a low pH or low pH-dependent host proteases, and that the cytoplasmic tail of XMRV Env critically modulates membrane fusion and cell entry. Our data also imply that additional cellular factors besides XPR1 are likely to be involved in XMRV entry.

## Introduction

Enveloped viruses must fuse with host cell membranes in order to gain entry and initiate infection. For retroviruses, this process is mediated by the envelope glycoprotein (Env) acquired from the viral producer cells. The Env is initially synthesized as a precursor in the endoplasmic reticulum (ER) and subsequently cleaved by cellular proteases in the *trans-Golgi* complex into the surface (SU) and transmembrane (TM) subunits [1]. The SU subunit contains a receptor binding domain (RBD) that is responsible for interactions with specific cellular receptors or coreceptors, and the TM subunit possesses a fusion peptide, two heptad repeats (HRs), a membrane-spanning domain (MSD), and a cytoplasmic tail (CT), all of which have been shown to control or regulate membrane fusion [2]. Upon proper triggering, the TM subunit undergoes a large scale conformational rearrangement, leading to the formation of a stable helix bundle (6-HB) that drives fusion between the viral and cellular membranes [3].

The retroviral Env-mediated fusion is controlled at multiple steps to prevent premature activation [2], [4]. First, the cleavage of retroviral Env precursor into SU and TM is a pre-requisite for fusion as it liberates the fusion peptide located at the amino terminus of TM so that it can insert into the target membrane upon triggering [3]. Second, post-translational modifications, such as glycosylation, are also critical for proper folding and receptor binding of Env thereby influencing membrane fusion and cell entry [5], [6], [7]. In addition, several retroviruses, such as murine leukemia virus (MLV), Mason-Pfizer monkey virus (M-PMV), equine infectious anemia virus (EIAV), etc, contain a ∼16 amino-acid stretch in the CT of Env, known as R peptide, that intrinsically restricts membrane fusion [8], [9], [10]. In the latter case, the Env proteins containing the full length CT are not fusogenic in the virus-producer cells, but become fully fusogenic after viral protease cleavage of the R peptide upon budding from host cells [9], [11], [12]. The mechanism underlying the R peptide-mediated control of retroviral Env fusion is still not known. Whereas fusion of most retroviruses is triggered by receptor binding, increasing numbers of retroviruses have been shown to require a low pH, or receptor binding plus low pH, for membrane fusion [13], [14], [15], [16], [17], [18], [19], [20]. It is interesting that infection by ecotropic murine leukemia virus (E-MLV) has been shown to be blocked by inhibitors of cellular cathepsins [21], suggesting host proteases are involved in the fusion activation of E-MLV and perhaps of other retroviruses. Similar mechanisms have been reported for other enveloped viruses [22], [23], [24], [25], [26].

Xenotropic murine leukemia virus-related virus (XMRV) is a gammaretrovirus that was originally identified from human prostate cancer patients and subsequently linked to chronic fatigue syndrome (CFS) [27], [28]. However, recent studies have shown that this virus is a recombinant mouse retrovirus that was likely generated during the passages of a human prostate tumor in nude mice [29], [30]. Moreover, numerous groups have failed to detect XMRV from human prostate cancer samples as well as CFS patients, making the claim of its association with these human diseases questionable [31], [32]. Regardless, it is still important to understand how the Env protein of XMRV mediates membrane fusion and cell entry from the virology perspective, especially in light of the emerging diverse mechanisms of retroviral Env-mediated fusion activation and cell entry [2]. The Env of XMRV shares significant sequence homology with that of other xenotropic and polytropic MLVs (X/P-MLV), especially in the SU subunit, and these viruses share the same xenotropic and polytropic retrovirus receptor 1 (XPR1) for entry [27], [33], [34], [35], [36]. XMRV has been shown to infect a wide range of cell lines derived from different species including humans, with the notable exception of hamster and mouse cells; overexpression of XPR1 in NIH 3T3 and CHO cells renders these cells susceptible to XMRV infection, indicating that XPR1 is the key cellular receptor for XMRV [37], [38], [39], [40], [41]. In this study, we aimed to understand the mechanisms of membrane fusion and cell entry mediated by the XMRV Env protein, particularly the possible role of its relatively long CT (compared to Mo-MLV) and of the viral receptor, XPR1, in modulating this process.

## Results

### XMRV entry is pH-independent and does not require cellular proteases

**Figure 1:**
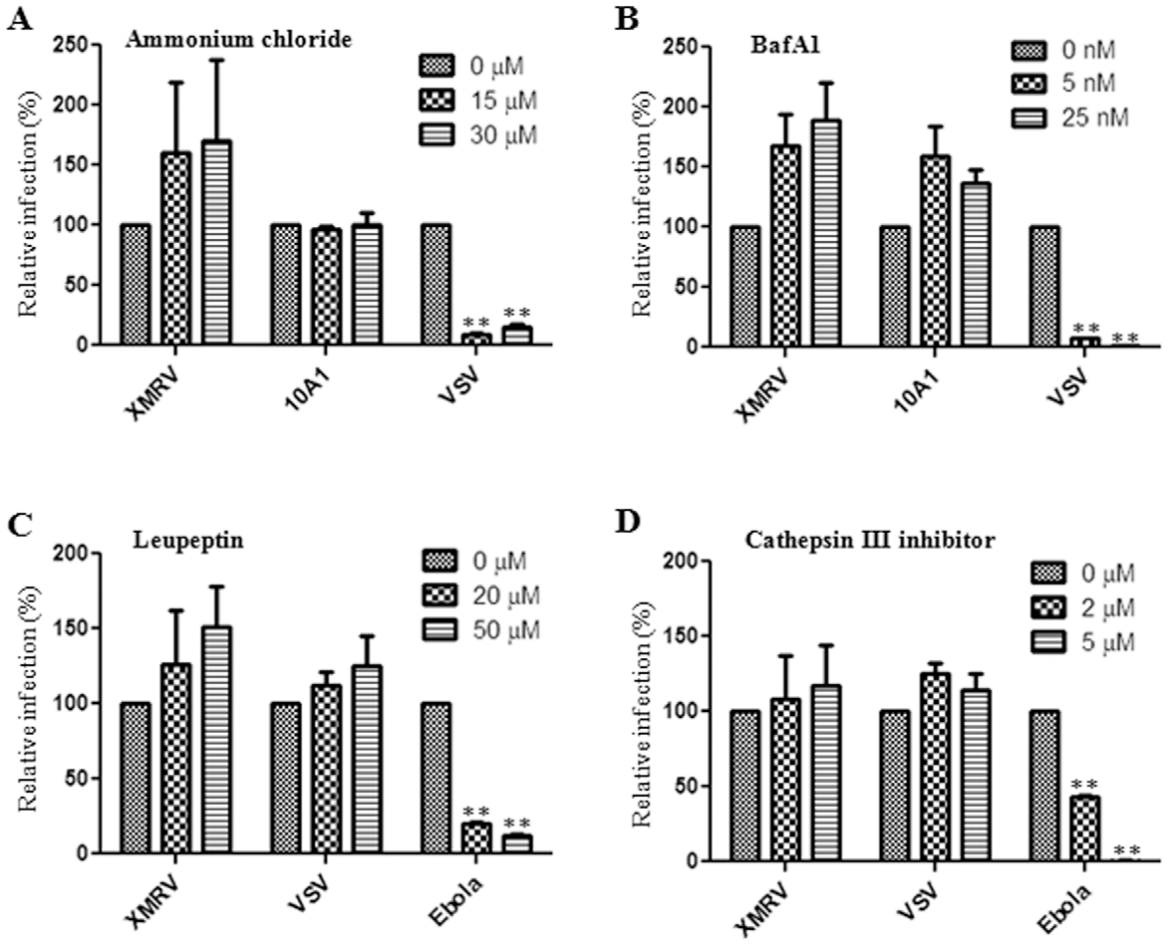
XMRV entry is pH-independent and does not require host proteases. HTX cells were pre-treated with indicated concentrations of (A) ammonium chloride, (B) Bafilomycin A1 (BafA1), (C) leupeptin or (D) cathepsin III inhibitor for 1 h and infected with MoMLV pseudotypes encoding GFP and bearing XMRV Env, MLV 10A1 Env, VSV-G or Ebola GP in the presence of drugs for 6 h before inactivation of remaining virions using citrate buffer. Percentage of GFP-expressing cells was measured by flow cytometry 48 h post-infection and normalized to the infection obtained in the absence of drug set to 100%. Shown are at least the averages of 3 independent experiments ± S.D. ** indicates p<0.01.

Retroviruses have been historically believed to fuse directly at the plasma membrane of target cells for entry and infection [42]. However, recent studies have shown that some retroviruses, including avian sarcoma leukosis virus (ASLV), mouse mammary tumor virus (MMTV), Jaagsiekte sheep retrovirus (JSRV), enzootic nasal tumor virus (ENTV), foamy virus, EIAV, and ecotropic Moloney MLV (MoMLV) require a low pH or low pH-dependent proteases for cell entry [13], [14], [15], [16], [17], [18], [19], [20], [21]. Here, we produced MoMLV pseudotypes bearing XMRV Env, and investigated the cell entry of XMRV by using classical chemical inhibitors that block pH-dependent viral entry [4]. We first treated human HTX cells (a subclone of HT1080) with a lysosomotropic agent, NH4Cl, and observed that it did not inhibit but rather somewhat enhanced XMRV infection (p>0.05). As expected, the infection of pH-dependent vesicular stomatitis virus (VSV) pseudovirions was dramatically decreased (p<0.01, Fig. 1A). We next treated cells with a proton-pump inhibitor, Bafilomycin A1 (BafA1), and found interestingly that XMRV infection was again increased (p>0.05), yet that VSV entry was almost completely blocked by BafA1 even at the 5 nM concentration (p<0.01, Fig. 1B). We noted that entry of 10A1 MLV was also slightly enhanced by BafA1 (p>0.05), but the effect was not dose-dependent (Fig. 1B). Similar effects of NH4Cl and BafA1 on XMRV entry were also observed in 293 and a human prostate cancer cell line, DU145 (data not shown), together supporting the idea that XMRV entry does not require a low pH as do the typical pH-dependent viruses, such as VSV and influenza A [4].

The modest but reproducible enhancement of XMRV infection in the presence of NH4Cl and BafA1 could be explained by a block of viral particle degradation in the endosomes or lysosomes. To investigate this possibility and explore if XMRV entry requires cellular proteases, we performed pseudoviral infection in the presence or absence of leupeptin or cathepsin III inhibitor, both of which are broad spectra, lysosomal protease inhibitors. XMRV infection was enhanced by both protease inhibitors, albeit the increase was not statistically significant (p>0.05); however, infection of Ebola pseudovirions was dramatically impaired (p<0.01, Fig. 1C and 1D). We noted that VSV infection was also slightly enhanced by these two protease inhibitors but the effect was not dose-dependent. The effect of these protease inhibitors on Ebola infection was consistent with the notion that Ebola GP-mediated membrane fusion with endosome requires cellular cathepsin B and L [22], [26]. Taken together, these results show that XMRV entry does not require a low pH or low pH-dependent cellular proteases, and that endocytosis XMRV may occur for XMRV but this would likely result in virions inactivation through pH-dependent host proteases.

### Creation of a soluble form of XMRV SU that binds to cells expressing viral receptor and blocks infection

**Figure 2:**
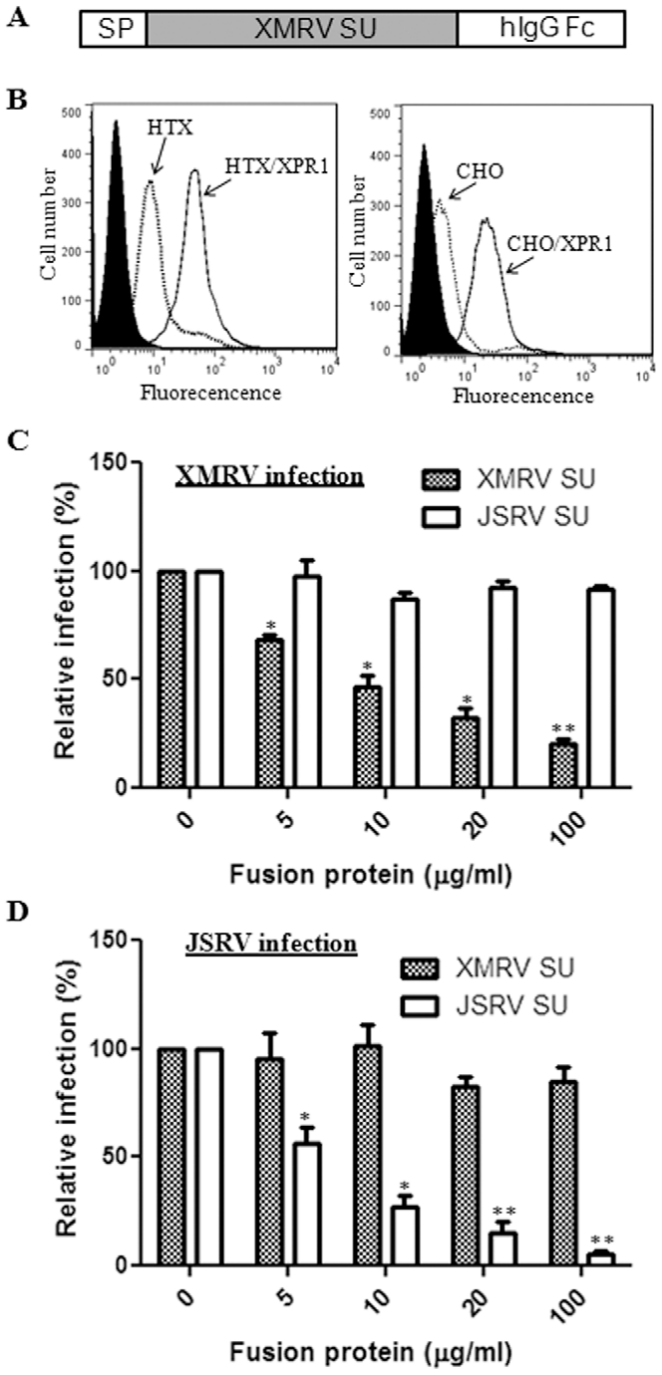
Soluble XMRV SU binds to cells expressing the viral entry receptor, XPR1, and blocks XMRV pseudovirion infection. (A) Schematic representation of XMRV SU-human immunoglobulin (hIgG) Fc fusion protein. SP: signal peptide. (B) Indicated cell lines were incubated with 5 µg of XMRV SU-hIgG Fc for 4 h and binding was measured using anti-human IgG FITC and flow cytometry. Filled: HTX or CHO cells stained with a FITC-labeled secondary antibody only (HTX/XPR1 or CHO/XPR1 cells gave a similar background signal which was not overlaid); broken line: parental HTX or CHO cells stained with XMRV SU plus secondary antibody; black line: HTX/XPR1 or CHO/XPR1 cells stained with both XMRV SU and secondary antibody. Representative experiments are shown (n = 5). (C, D) HTX cells were pre-bound with indicated amounts of soluble XMRV or JSRV SU for 1 h at 4°C and infected with MoMLV pseudotypes encoding alkaline phosphatase (AP) and bearing XMRV Env (C) or JSRV Env (D) for 6 h before inactivation of remaining virions using citrate buffer. AP foci were counted 72 h post-infection and values were normalized to infection in the absence of soluble protein set to 100%. Shown are the averages of 3 independent experiments ± S.D. * indicates p<0.05; ** indicates p<0.01.

In order to investigate the role of interactions between XMRV Env and its receptor XPR1 in modulating membrane fusion and cell entry of XMRV, we created a soluble form of XMRV SU fused to the human IgG Fc fragment (Fig. 2A). The fusion protein was produced by transient transfection of 293T cells and purified using protein A beads using a procedure we had previously described for the JSRV SU fusion protein [43]. As shown in Figure 2B, incubation of XMRV SU-human IgG fusion protein with the permissive HTX cells resulted in an apparent fluorescence shift relative to that of secondary antibody alone (which served as a negative control), and overexpression of XPR1 receptor in HTX cells substantially increased the XMRV SU binding to the cells, indicating that the binding was specific. Similar results were also obtained in the permissive human 293, DU145, A549, dog MDCK, and monkey Vero cells (data not shown). The specific binding of XMRV SU for XPR1 was further confirmed in CHO/XPR1 cells which were established by transduction using a retroviral vector expressing XPR1; but surprisingly, we reproducibly detected a fluorescent shift in the parental CHO cells (Fig. 2B), which are known to be nonpermissive for XMRV infection [37], [41] (also see Table 1 below).

**Table 1:** Titers of XMRV Env pseudovirions in CHO and CHO/XPR1 cells.

Construct	Titer (AP^+^ FFU/ml)	
	CHO	CHO/XPR1
XMRV Env	<2	2×10^4^
CT635	<2	4×10^3^
CT624	<2	2×10^3^
CT613	<2	1×10^2^
CT609	<2	50
CT608	<2	34
CT606	<2	<2

293/GP-LAPSN cells expressing MoMLV Gag-Pol and alkaline phosphatase (AP) were transfected with plasmids encoding individual XMRV Envs. Virions were harvested 48–72 h post-transfection, and used to infect CHO and CHO/XPR1 cells expressing XPR1. Titers were determined by counting AP^+^ foci 72 h post-infection. Results of a representative experiment are shown. Experiments were repeated three times, with similar titers obtained.

We next assessed the effects of purified XMRV SU fusion protein on pseudoviral infection in HTX cells. Cells were pre-incubated with different amounts of XMRV SU fusion proteins for 1 h at 4°C, followed by switching the temperature to 37°C to initiate infection in the constant presence of the fusion protein for 6 h. As shown in Figure 2C, the XMRV SU fusion proteins substantially blocked the XMRV pseudoviral infection (p<0.05) in a dose-dependent manner, with the JSRV SU having no apparent effect (p>0.05). As would be expected, the JSRV SU fusion protein specifically blocked the JSRV pseudoviral infection but not that of XMRV (p<0.05) (Fig. 2C and 2D). The concentration of soluble XMRV SU required to block 50% of XMRV infection was ∼10 ug/ml, which was relatively higher compared that of JSRV SU (∼5 µg/ml, which is necessary to block 50% of JSRV infection) (Fig. 2C and 2D). Together, these results demonstrate that the soluble XMRV SU fusion protein interacts with the XPR1 receptor on the cell surface and functionally blocks the XMRV pseudoviral infection.

### Truncation of XMRV Env from the C-terminal cytoplasmic tail (CT) promotes SU shedding and syncytia-forming activity

**Figure 3:**
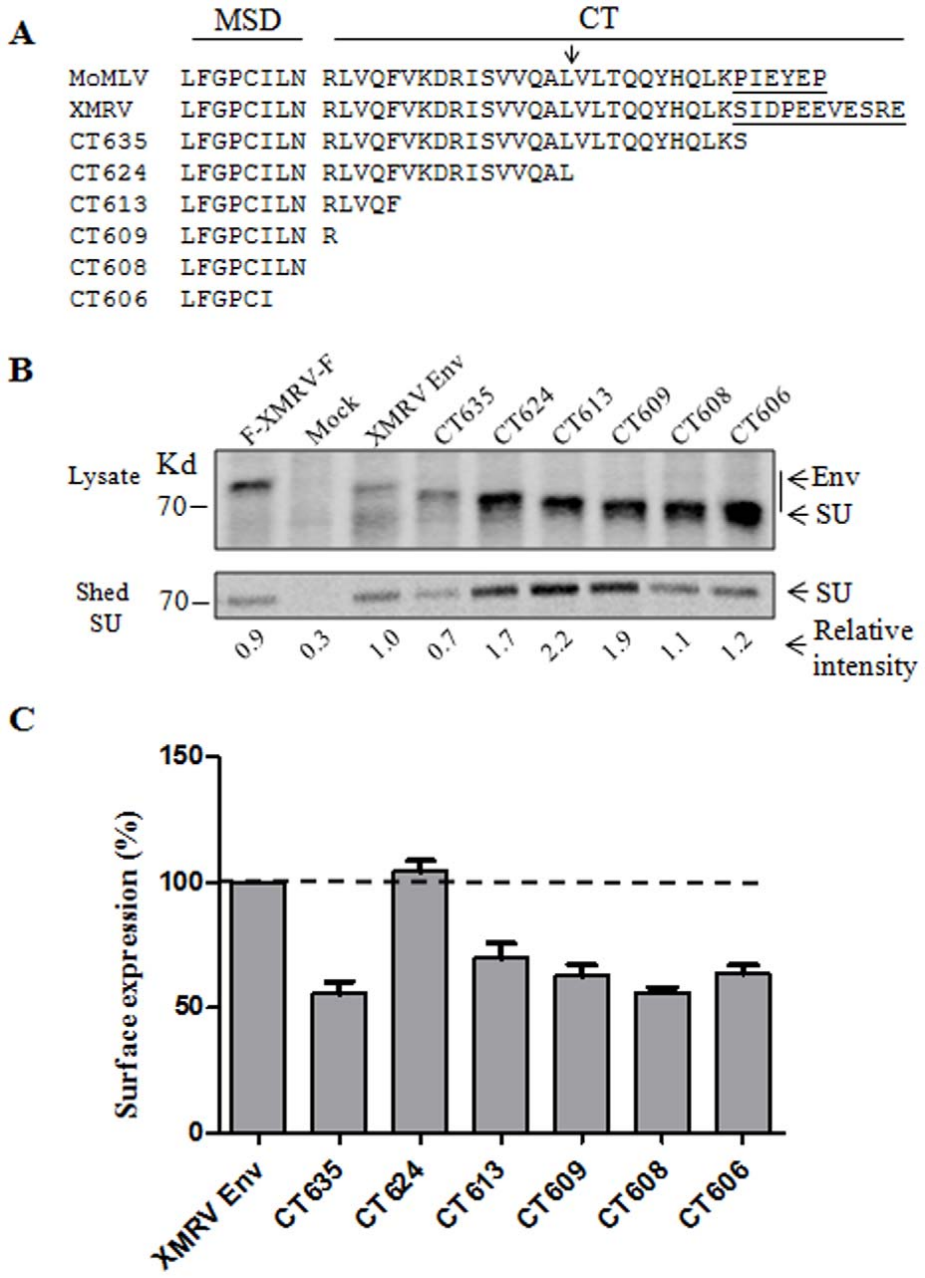
C-terminal truncation of XMRV Env induces SU shedding. (A) Sequence alignment of MoMLV and XMRV Env constructs. MSD: membrane spanning domain. CT: cytoplasmic tail. Arrow: indicates the MoMLV Env R-peptide cleavage site. Underlined: The CT region that differs between XMRV and MoMLV. (B) 293T cells expressing XMRV Env were metabolically labeled for 1 h and chased for 4 h. Env proteins in cells lysates and shed in the culture media were immunoprecipated, resolved by SDS-PAGE and subjected to autoradiography. Band density was measured using the Quantity One software and values were normalized to the intensity of the XMRV Env SU set to 1.0. Representative experiment is shown (n = 2). (C) The expression of XMRV Env on the 293T cell surface was measured using anti-FLAG and flow cytometry. Fluorescence geometric means were normalized to XMRV Env (100%). Shown are the averages of 3 independent experiments ± S.D. XMRV Env: XMRV Env tagged with a FLAG sequence only at the N-terminus. All truncations were also tagged similarly with an N-terminal FLAG. F-XMRV-F: an XMRV Env construct that is tagged by FLAG sequences on both N- and C-termini. Mock: untransfected 293T cells.

While identical in the N-terminal and central regions, including the conserved R peptide cleavage site between 624 and 625, the C-terminal CT of XMRV Env differs from that of MoMLV Env, with a relatively longer length (Fig. 3A). Here we sought to determine the membrane fusion property of XMRV Env, particularly the effect of CT truncation on cell fusion. We first created a series of truncation mutants in the CT and examined the Env processing and expression by metabolic labeling. 293T cells were pulse-labeled with [^35-^S] Met-Cys for 1 h and chased for 4 h; the XMRV Env proteins in the cell lysates and their SU shed into the culture media were immunoprecipitated with anti-FLAG beads (FLAG is tagged at the N-terminus of SU). As shown in Figure 3B, all the Env constructs were properly processed and expressed in the transfected cells, except CT635 which consistently showed a decreased level of expression of the processed SU (∼30% of wildtype) (note the SU subunits of CT624, CT613, CT609, CT608 and CT606 co-migrated with their full length precursors because of their reduced size of precursor, Fig. 3B, upper panel). Of note, CT624, CT613 and CT609 exhibited enhanced SU shedding into culture media as compared to that of wildtype and other mutants (Fig. 3B, lower panel). We also examined the SU surface expression of these Env constructs in 293T cells by flow cytometry using an anti-FLAG antibody, and observed that CT624 exhibited a wildtype level of expression whereas all the other truncation mutants had reduced SU on the cell surface (∼50%) (Fig. 3C). Altogether, these results demonstrate that truncation of the CT of XMRV Env affects SU shedding and surface expression.

**Figure 4:**
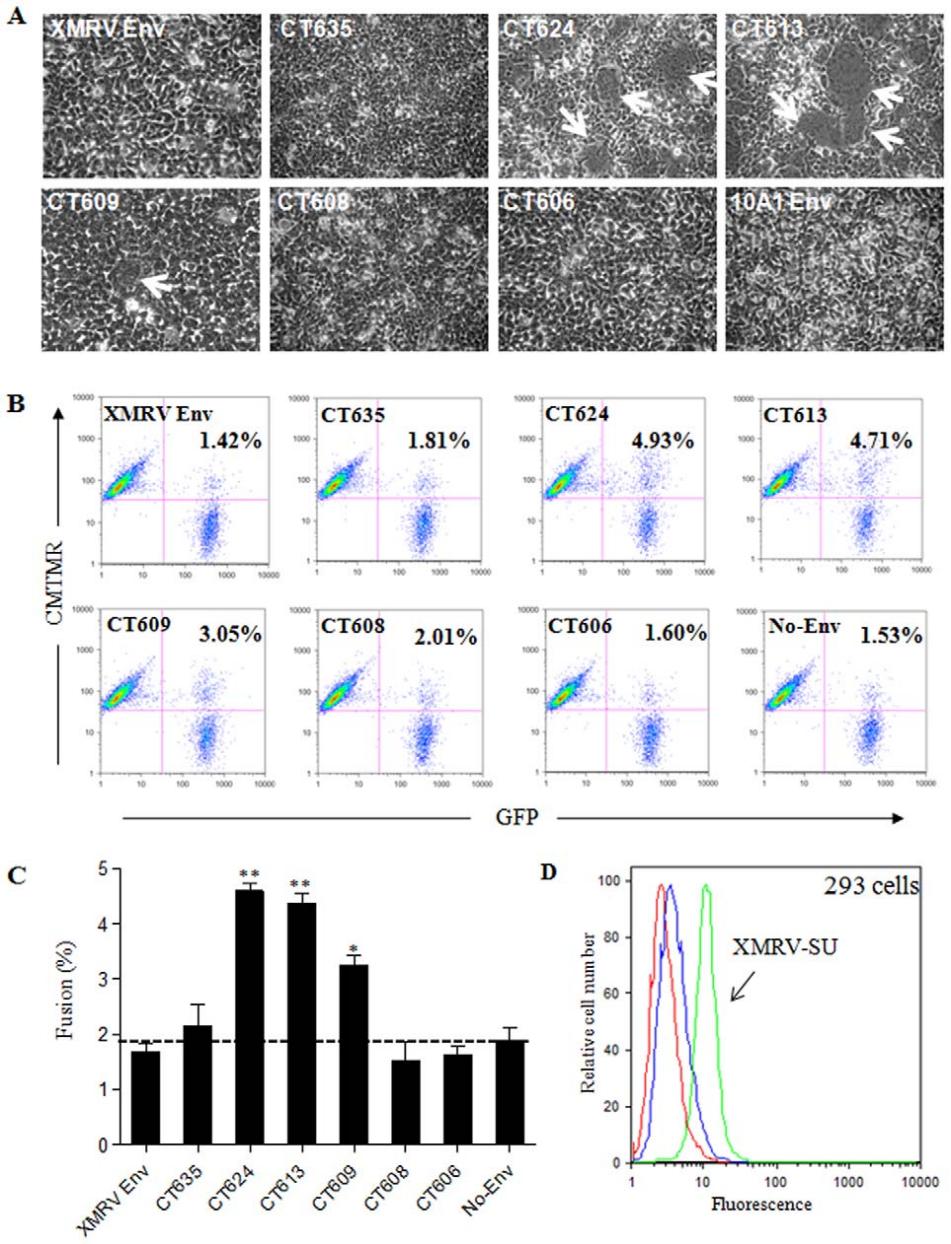
C-terminal truncation of XMRV Env in the CT activates its fusion activity. (A) Syncytium-induction assay. 293 cells were transiently transfected with plasmids encoding the indicated XMRV Env and photographed 24 h post-transfection. Arrows indicate syncytia. (B) Cell-cell fusion. Effector 293T-GFP cells expressing indicated XMRV Env or no envelope (No Env) were co-cultured for 6 h with CMTMR-labeled target 293 cells and analyzed by flow cytometry. Values shown represent the percentages of fused cells. (C) The fusion percentages (GFP^+^/CMTMR^+^) of 3 independent experiments performed in duplicate were averaged (±S.D.) and plotted. (D) Binding of XMRV SU to target 293 cells. XMRV SU (2 µg) was bound to cells for 4 h at 4°C, stained with anti-human IgG FITC, and analyzed by flow cytometry. Red: unstained cells. Blue: secondary alone. Green: XMRV SU and secondary. A representative experiment is shown (n = 4).

We next performed syncytia-forming assay in 293 cells and assessed the membrane fusion properties of XMRV Env and mutants. 293 cells were chosen because they are highly transfectable and also permissive to XMRV infection [41]. The full length XMRV Env was unable to induce syncytia formation, presumably due to the presence of an R peptide in the CT (Fig. 4A). CT624 and CT613, in which the CT of XMRV Env was truncated at the putative R peptide cleavage site and further towards the N-terminus (Fig. 3A), respectively, elicited apparent syncytia (typically ∼30 syncytia per µg DNA, with >6 nuclei per syncytium) (Fig. 4A). Interestingly, CT609, which contains the first arginine residue of the CT only, showed a much reduced fusion activity as compared to CT624 and CT613 (∼5–10 syncytia per µg DNA, with smaller size) (Fig. 4A). These results were somewhat different from what had been reported for MoMLV, where an identical mutant largely retained the fusogenicity of R peptide-minus mutant [11], [44]. Noticeably, the increased fusion activity of CT624 and CT613, and to lesser extent of CT609, correlated with the enhanced SU shedding of these mutants in culture media (Fig. 3B). These results are similar to our previous findings made on JSRV Env, severe truncation of which led to pronounced SU shedding accompanied with greatly increased fusogenicity [19]. Interestingly, we observed that the tailless CT608 and CT606 mutants were virtually fusion-defective, possibly due to their truncation into the MSD and/or reduced surface expression (Fig. 3A and 3C). We further treated the individual Env-expressing cells with a low pH buffer (pH 5.0) for 1 min or 5 min (pictures not shown), but did not observe apparent effect on syncytia induction of any of these constructs, supporting the above conclusion that XMRV Env-mediated fusion and cellular entry is pH-independent.

### Severe truncation of XMRV Env proximal to the membrane-spanning domain impairs fusogenicity

The finding that syncytia induction can be observed in cells expressing XMRV Env truncation mutants prompted us to further quantitatively measure their membrane fusion activities using a flow cytometry-based cell-cell fusion assay adapted from our previous studies on JSRV [18], [19], [45]. In this assay, the effector 293T/GFP cells were transfected with Env-encoding plasmids, and the target 293 cells were labeled with a red-fluorescent dye, CMTMR. Consistent with the syncytia formation data, the XMRV Env wildtype and CT635 were unable to induce cell-cell fusion as evidenced by no fluorescent dye transfer (∼1.8%, similar to the No-Env background), whereas truncation at the putative R-peptide cleavage site or further upstream towards the MSD, i.e., CT624 or CT613, induced apparent cell-cell fusion (4∼5%) (p<0.05) (Fig. 4B and 4C). Again, CT609 showed a relatively low cell-cell fusion activity (∼3%), and the tailless CT608 and CT606 mutants were incapable of inducing fusion with background signals (Fig. 4B and 4C). We also examined the fusion activities of all constructs in 293 cells overexpressing the XPR1 receptor (293/XPR1), but observed only modest increases for CT624, CT613 and CT609 (∼10–20%). The titers of XMRV wildtype and mutants in 293/XPR1 cells were approximately 5-fold higher than those in the parental 293 cells, and the overexpression of XPR1 in 293/XPR1 cells was confirmed by flow cytometry using the soluble XMRV SU (data not shown).

**Figure 5:**
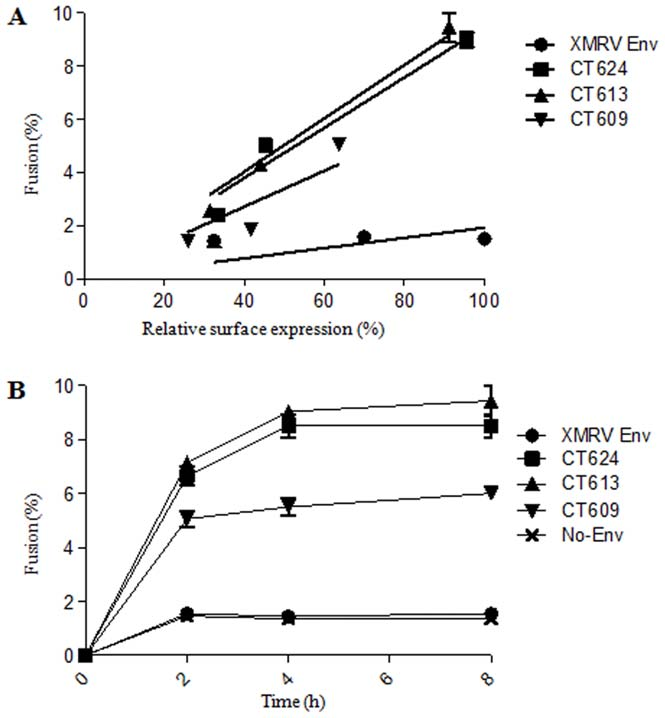
Truncation beyond the putative R-peptide cleavage site does not further enhance the XMRV Env fusion activity. (A) Effect of Env expression on cell-cell fusion. Effector 293T/GFP cells were transfected with different amounts of plasmids encoding indicated XMRV Env, and co-cultured with target CMTMR-labeled 293 cells. Fusion percentages were determined by flow cytometry following 6 h incubation. In parallel, the XMRV Env surface expression was measured by flow cytometry using an anti-FLAG antibody. The obtained fusion percentages were plotted against Env surface expression. A representative experiment with standard errors of triplicate samples is shown (n = 3). (B) Effect of incubation time on cell-cell fusion. Effector 293T/GFP cells expressing indicated XMRV Env were co-cultured with target CMTMR-labeled 293 cells and cell-cell fusion was assessed after different periods of incubation time. A representative experiment with standard errors of triplicate sample is shown (n = 3).

The differential fusion activities of CT624, CT613 and CT609 could be due to their different levels of Env expression on the cell surface or/and intrinsic fusogenicity. To distinguish these possibilities, we transfected effector 293T cells with different amounts of plasmid DNA encoding individual truncated Envs, and determined their cell-cell fusion activities and SU surface expression in parallel. As shown in Figure 5A, the fusion profiles of CT624 and CT613 were almost identical, as evidenced by their similar slopes (∼0.095 and ∼0.010, respectively, R^2^ = 0.97–0.99). In contrast, CT609 exhibited a slightly decreased slope (∼0.068, R^2^ = 0.93), implying that its reduced fusogenicity relative to CT624 and CT613 cannot be fully attributable to its low level of surface expression. We further performed cell-cell fusion using different co-culture periods, i.e., 0, 2, 4, and 8 h, and again observed faster fusion kinetics for CT624 and CT613 (Fig. 5B) as compared to CT609, further confirming that additional truncation of XMRV Env beyond the R peptide cleavage site does not increase fusion activity as we had seen for JSRV Env and that CT609 has an intrinsically relatively low fusogenicity.

To examine the possibility that the lack or reduced fusion for CT609, CT608 and CT606 might be due to a block at hemifusion, we treated co-cultured target and effector cells with chlorpromazine (CPZ, 0.2–0.5 mM), a membrane permeable reagent that promotes the transition from hemifusion to full fusion [46], but observed no apparent increase in fusion for any of these Env constructs (data not shown). These results suggest that the fusion suppression in these XMRV Env constructs unlikely takes place at the hemifusion step. Overall we conclude that, distinct from JSRV Env, severe truncation of the CT of XMRV Env towards the MSD does not further enhance but rather impairs the Env fusogenicity. The reason for the decreased fusogenicity of CT609, CT608 and CT606 remains unclear, but is likely related to reduced surface expression or/and altered Env conformation (see Discussion).

### Truncation of XMRV Env causes membrane fusion in nonpermissive CHO cells but does not confer pseudoviral infection in the same cell type

**Figure 6:**
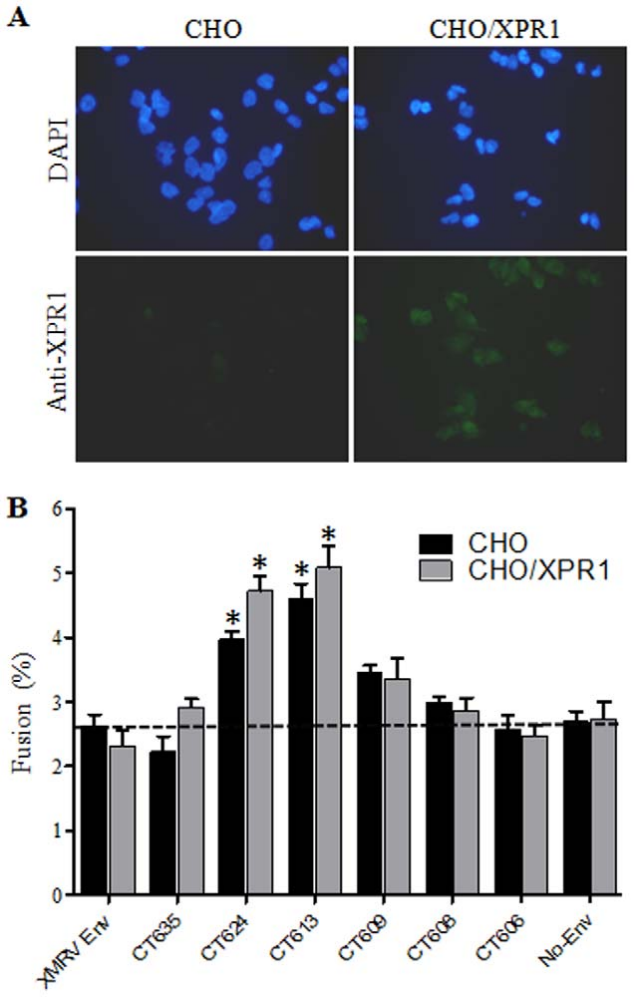
Cell-cell fusion activities of XMRV Env and mutants in CHO or CHO cells expressing XPR1. (A) Immunostaining of CHO and CHO/XPR1 cells. Cells were fixed, permeabilized, stained for XPR1 using anti-XPR1 and FITC-coupled secondary antibodies, and counterstained using DAPI. (B) XMRV Env truncation mutants can induce cell-cell fusion in non-permissive CHO cells. Effector 293T/GFP cells expressing indicated XMRV Env were co-cultured for 6 h with target CMTMR-labeled CHO or CHO cells expressing XPR1 (CHO/XPR1) and analyzed for fusion by flow cytometry. Shown are the averages of 3∼6 independent experiments ± S.D. * indicates p<0.05.

We next determined the role of XPR1 in XMRV Env-induced membrane fusion by using nonpermissive CHO cells and CHO cells expressing human XPR1 (CHO/XPR1). The CHO/XPR1 cell line was established by transducing CHO cells with a LXSN retroviral vector encoding XPR1 [33]. Expression of XPR1 in the CHO/XPR1 cell line was demonstrated by the specific binding of soluble XMRV SU fusion protein to those cells as shown in Figure 2B, and was further confirmed by immunostaining using an anti-XPR1 antibody (Fig. 6A). The titers of XMRV wildtype and CT truncation mutants in these two cell lines are shown in Table 1. CHO cells were apparently not susceptible to XMRV Env pseudoviral infection (Table 1), consistent with previous reports from other groups [38], [39], [40], [47]. Overexpression of XPR1 in CHO cells resulted in a titer of 10^4^ IU/ml for the wildtype and somewhat reduced titers for the CT truncation mutants (Table 1). Overall, these data support the notion that XPR1 is a critical cellular receptor for XMRV.

The cell-cell fusion activities of XMRV Env and mutants in CHO and CHO/XPR1-expressing cells were then examined. For this purpose, we labeled CHO or CHO/XPR1 cells with CMTMR, and co-cultured them with the effector 293T/GFP cells expressing XMRV Env or truncation mutants plus GFP. We observed that, surprisingly, CT624 and CT613 reproducibly induced a detectable level of cell-cell fusion activity in the non-permissive CHO cells (p<0.05) (Fig. 6B), despite the fact that this cell line is nonpermissive for XMRV infection (Table 1). Interestingly, overexpression of human XPR1 in CHO cells only slightly increased the fusion activities of XMRV Env CT mutants, CT624 and CT613 (Fig. 6B), despite their significantly increased pseudoviral titers (Table 1). These results, along with the data using 293/XPR1 cells described above, imply that XPR1 may not be the sole trigger for XMRV Env-mediated membrane fusion and cell entry.

### Incorporations of XMRV Env into retroviral and lentiviral vectors are impaired by CT truncations resulting in reduced transduction efficiency

**Figure 7:**
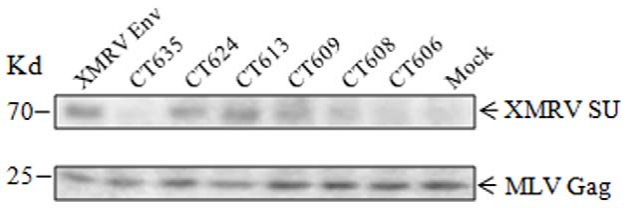
XMRV Env incorporations into an MLV retroviral vector. 293/GP_LAPSN cells were transfected with an XMRV Env-encoding plasmid, and viral particles were purified through ultracentrifugation. Concentrated viruses were subjected to Western blot using an anti-FLAG antibody to detect XMRV SU (upper panel) or an anti-MLV gag antibody to examine MLV Gag (lower panel).

The CT of retrovirus Env plays various roles in the replication cycle, including entry and assembly; this has been mostly studied in HIV-1 [48]. Here, we wished to determine the ability of XMRV Env and CT truncation mutants to pseudotype the MoMLV retroviral and HIV-1 lentiviral vectors as well as its relationship to membrane fusion and cell entry. As shown in Table 2, all the XMRV Env constructs (tagged with a FLAG sequence at the N-terminus) were able to pseudotype both types of vectors but with distinct efficiencies. The full length XMRV Env exhibited approximately 2×10^4^ infectious units per ml for both vectors (Table 2, and data not shown), similar to a recent report [49]. The titers of MoMLV retroviral pseudotypes harbouring CT635 or CT624 were slightly reduced as compared to that of the wildtype Env (∼4–6 fold), whereas the other more severely truncated mutants exhibited a 2∼3-log decrease in the infectious titer (Table 2). Similar patterns were also observed for the HIV-1 lentiviral pseudotypes (Table 2), but interestingly we found that CT635 consistently exhibited pronounced reductions in the lentiviral pseudoviral titers (∼100 fold) as compared that of MoMLV retroviral pseudotypes (∼6-fold). The generally reduced viral titers for the CT truncations cannot be fully explained by the enhanced SU shedding, at least for some of these mutants, but appeared to correlate with the differential levels of SU surface expression (Fig. 2C). We also examined the incorporation efficiencies of these Envs into the MLV pseudovirions by Western blot using concentrated pseudoviral particles, and detected similar levels of SU for CT624, CT613, CT609 and the wildtype Env, as compared to CT635, CT608 and CT606 for which the SU incorporation efficiency was greatly reduced (Fig. 7). We have attempted to detect the XMRV TM in viral producer cells and the viral particles using an antibody against the MoMLV TM but without success (a gift from Marc Johnson, data not shown). Nevertheless, the Env incorporation data based on the SU (Fig. 7) and the XMRV pseudotype titers shown in Table 2 correlated with the SU expression profiles shown in Figure 3C. We noticed that the titers of pseudoviral infection for CT624 and CT613 did not correlate with their enhanced Env fusogenicity based on the syncytia formation and cell-cell fusion assays, and this was particularly the case for CT613, which showed a strongly enhanced fusogenicity (Figs. 4 and 5) but a much reduced titer relative to the wildtype Env (Tables 1 and 2).

**Table 2:** Titers of MLV and HIV-1 vectors bearing XMRV Env and truncation mutants.

Construct	MLV vector(AP+ FFU/ml)	HIV vector(IU/ml)
XMRV Env	5.1×10^4^	1.6×10^4^
CT635	8.4×10^3^	1.8×10^2^
CT624	1.4×10^4^	6.2×10^3^
CT613	7.9×10^2^	4.4×10^2^
CT609	4.0×10^2^	2.8×10^2^
CT608	8.8×10^2^	2.3×10^2^
CT606	4.1×10^2^	50

The MLV titers were determined as described in Table 1 except HTX cells were used for infection. For HIV-1 lentiviral vectors, 293T cells were transfected with a plasmid encoding HIV-1 Gag-Pol, an HIV-1 vector expressing GFP and a plasmid expressing XMRV Env or truncation mutant. Virions were harvested 48–72 h post-transfection, and used to infect HTX cells. Titers were determined by detecting GFP^+^ cells 48 h post-infection. Results of a typical representative experiment are shown. Experiments were repeated three times, with similar titers obtained.

## Discussion

Retroviruses use distinct mechanisms for membrane fusion and cell entry, the mechanisms of which are still poorly defined. In this report, we provided evidence that XMRV entry does not require a low pH or pH-dependent host proteases, but uses a mechanism that is similar to that of typical pH-independent viruses. Interestingly, we find that XMRV entry is enhanced by NH4Cl and BafA1, the two most commonly used agents that neutralize acidic endosomal environments, as well as by leupeptin and cathepsin inhibitor III, which broadly inhibit the lysosomal protease activities. Together, these observations suggest that endocytosis may occur in non-productive entry of XMRV, leading to viral particle degradation. Consistent with this notion, we did not observe specific block of XMRV entry by Dynasore or a dominant negative mutant of Dynamin (K44A) in 293T cells (data not shown). Previous studies have shown that different endocytic pathways mediate entry of some pH-independent retroviruses, including amphotropic and ecotropic MLV as well as HIV-1 [21], [50], [51], however the exact mechanisms and the underlying significance remain largely unknown. It should be added that, while we have not observed any inhibitory effects of leupeptin and cathepsin III inhibitor on XMRV infection in HTX and 293 cells, we cannot rule out the possibility that cellular proteases may be involved in the XMRV entry of other cell types. In this sense, it is interesting to note that endocytosis and cathepsins were recently shown to affect the entry of several gammaretroviruses, including XMRV, in human TE671 and rat XC cells [52]. Additional studies are warranted to clarify this issue and further characterize the entry pathway of XMRV, perhaps with assistance of the recently developed single molecule labeling and confocal imaging technique.

One important objective of this study was to understand the possible roles of the CT of XMRV Env in modulating membrane fusion and cell entry. We showed here that CT624 and CT613, which are truncated at or beyond the putative R peptide cleavage site of the XMRV Env (Fig. 3A), induced apparent syncytia formation and cell-cell fusion in permissive 293 cells (Figs. 4 and 5), presumably due to the removal of the putative R peptide. Surprisingly, we observed apparent cell-cell fusion of CT624 and CT613 also in CHO cells (Fig. 6B), which are known to be non-permissive for XMRV infection, including these two truncation mutants (Table 1). These results suggest two possibilities: first, CHO cells may express a low but functional level of XPR1 that permits cell-cell fusion of XMRV Env mutants and that the resistance of CHO cells to XMRV infection may be due to a block at the post-fusion steps. This possibility is supported in part by our observation that a soluble form of XMRV SU fusion protein reproducibly binds CHO cells relative to the negative control using secondary antibody alone (Fig. 2). These binding results also argue against the possibility that potential N-linked glycosylation of XPR1 in CHO accounts for its resistance to XMRV infection, a situation that has been previously shown to be the case for several retroviruses [53], [54]. Second, the XPR1-mediated binding may not be the sole trigger for XMRV Env-mediated membrane fusion. This scenario is in line with our finding that overexpression of XPR1 in 293 and CHO cells did not significantly increase the cell-cell fusion activities of XMRV Env truncation mutants despite their increased infection in these cells (Fig. 6B and Table 1; data not shown). We also considered the possibility that the XMRV Env truncation mutants may have acquired a receptor-independent, spontaneous cell-cell fusion or are pre-activated in 293T/GFP cells due to their reduced kinetic barrier required for membrane fusion; however, the lack of infection in the CHO cells for the truncation mutants did not support this hypothesis (Table 2). Taken together, we favour the notion that, while XPR1 is a critical receptor for XMRV and is required for membrane fusion and cell entry, other cellular factors as yet to be identified are likely to be involved in cell entry and membrane fusion of XMRV. Consistent with this idea, it has been recently reported that XMRV does not infect BHK cells even when XPR1 is overexpressed in this cell line [47], and that XMRV can infect A549 cells even though this cell line does not express a functional XPR1 receptor [55]. Hence, identification of additional cellular factors involved in XMRV entry would help to better understand the mechanisms of membrane fusion and cell entry mediated by XMRV Env.

Previous studies from HIV and other simple retroviruses have suggested that the enhanced fusion activities of some retroviral Env truncations in the CT may be due to increased steady-state levels of Env expression on the cell surface [56], [57], [58], [59]. However, here we have found little evidence that suggests that this might be the case for XMRV (Fig. 3C) - despite the highly conserved endocytosis motif, YXXθ (Y = tyrosine, X = any amino acid, θ = residue with hydrophobic side chain) present in the CT of XMRV Env (Fig. 3A). Another commonly assumed mechanism is that truncation of the retroviral Env CT can somehow alter the conformation of Env ectodomain, resulting in a reduced association between SU and TM thereby promoting membrane fusion [60]. Indeed, we observed that all three truncation mutants with enhanced fusogenicities, i.e., CT624, CT613 and CT609, exhibited increased levels of SU shedding, which was in sharp contrast to that of wildtype Env and other mutants (CT635, CT608 and CT606) having minimal cell-cell fusion activity (Fig. 3B, Figs. 4 and 5). Future studies will focus on how the CT of XMRV Env structurally modulates the Env fusion activation.

Another surprising finding of this study is that CT609, which harbours the single arginine residue in the CT possesses a reduced fusogenicity relative to that of CT624 and CT613, which cannot be solely explained by its reduced surface expression (Figs. 4, 5, and 6). This observation is clearly different from what we had seen for JSRV Env [19] and is also somewhat different from some though not all of the previous studies on MoMLV [11], [44], [61]. Importantly, the tailless mutants, CT608 and CT606, are virtually fusion-defective (Fig. 4), collectively leading us to propose that the N-terminal CT proximal to the MSD of XMRV Env is critical for Env-mediated membrane fusion. One possible mechanism is that residues in this region, including the highly conserved arginine present in many transmembrane proteins including the retroviral Envs, may interact with cell membrane and thus modulate lipid movement during the membrane fusion process [62], [63].

Despite enhanced fusogenicity of CT624 and CT613, we found that the infection efficiency of their pseudovirions were rather low compared to those of wildtype Env (Tables 1 and 2). One plausible explanation is that the incorporations of these truncations into the MoMLV and HIV-1 vectors might be reduced as compared to those of wildtype; however, based on our immunoblot analysis using an anti-FLAG antibody to detect the XMRV SU, we found no evidence to support this scenario (Fig. 7). Alternatively, the reduced pseudoviral titers for the truncation mutants might result from their altered ability to bind to the viral receptor, XPR1. While we do not have direct evidence in support of this possibility, the apparent SU shedding induced by the CT truncations in the Env-expressing cells (Fig. 3B) strongly suggests that conformational changes likely occurs in the ectodomain of the truncated Env, including their SU subunits. Indeed, prior studies from HIV and other retroviruses have demonstrated that CT truncation of retroviral Env can alter the Env receptor binding capability thus affect viral infection [60], [64]. In this regard, it would be interesting to explore the role of the CT of XMRV Env in the infectious virus system.

## Materials and Methods

### Cell lines, antibodies and reagents

The HTX (a subclone of HT1080), 293T, 293, 293T/GFP (293T cell line stably expressing GFP) and 293/GP-LAPSN (293 cells stably expressing MoMLV Gag-Pol and alkaline phosphatase or AP) cell lines have been previously described [19], [65]. The 293/XPR1, HTX/XPR1 and CHO/XPR1 cell lines were generated by transducing the 293, HTX or CHO cells using a retroviral vector, LXSN, encoding the XPR1 receptor (LhXPR1SN, kind gift of Dusty Miller) [33] and bearing VSV-G. Infected cells were selected using G418 (Invitrogen, Carlsbad, CA) for ∼10 days. All cell lines were cultured in DMEM (Invitrogen) supplemented with 10% fetal bovine serum (FBS) at 37°C at 10% CO_2_-air atmosphere at 100% relative humidity.

The anti-FLAG monoclonal antibody, the EZview Red anti-FLAG affinity gel, and anti-mouse immunoglobulin G (IgG) coupled to phycoerythrin (PE) were purchased from Sigma (St. Louis, MO). The secondary anti-human IgG antibody coupled to fluorescein isothiocyanate (FITC) was purchased from DAKO Cytometer (Glostrup, Denmark). The anti-XPR1 antibody was purchased from Abcam (Abcam, Cambridge, MA). The red fluorescent dye 5-(and-6)-([{4-chloromethyl}benzoyl}amino)tetramethylrhodamine (CMTMR) and Lipofectamine 2000 were purchased from Invitrogen (Carlsbad, CA). Ammonium chloride, chlorpromazine (CPZ), 4′,6-diamidino-2-phenylindole (DAPI) were purchased from Sigma (St. Louis, MO). Bafilomycin A1 (BafA1), leupeptin hemisulfate and cathepsin inhibitor III were purchased from Calbiochem (Darmstadt, Germany). The [^35^S] Methionine and [^35^S] Cysteine cell labeling pro-mix was purchased from Amersham (Buckinghamshire, England).

### XMRV Env constructs

XMRV Env was initially engineered to contain a FLAG tag at both N- and C-termini by using pcDNA3.1-VP62 (gift of Robert Silverman) [37] as a template for PCR, and cloned in a pCIneo expression vector (Promega, Madison, WI), the resulting construct was referred to as pCIneo-F-Xenv-F. To create the N-terminal FLAG-tagged XMRV Env wildtype and CT truncations, the pCIneo-F-Xenv-F construct was used as a template, with the following lower primers being used for PCR amplification (Not I sites are underlined): XMRV Env, 5′- ATCGGCGGCCGCTCATTCACGTGATTCCACTTC-3′; CT635, 5′- TTCTGCGGCCGCTCATGATTTGAGTTGGTGATA-3′; CT624, 5′-CTGTGCGGCCGCTCACAGGGCCTGCACTACCGA-3′; CT613, 5′-AATTGCGGCCGCTCAAAACTGGACCAAGCGGTTG-3′; CT609, 5′-TACAGCGGCCGCTCAGCGGTTGAGAATACAGGGTCCGA-3′; CT608, 5′- AAACGCGGCCGCTCAGTTGAGAATACAGGGTCCGA-3′; CT606, 5′- GACCGCGGCCGCTCAAATACAGGGTCCGAAGA-3′. The pCIneo-10A1, pMD.G and pCIneo-Ebola GP expression vectors have been previously described [18].

The soluble XMRV SU construct was generated by overlapping PCR using pcDNA3.1-VP62 [37] and the previously described pCSI-JSU (for JSRV SU fusion protein) [43] as templates. The first fragment containing XMRV SU was amplified using the following primers: upstream primer (Not I underlined), 5′- GCATGCGGCCGCATGGAAAGTCCAGCGTTCTC-3′; downstream primer, 5′-CCTAGGCCTGTCGACGCCTTTTCAAACTGGCC-3′. The second fragment containing human IgG Fc was generated using the following primers: upstream primer, 5′- GGCCAGTTTGAAAAGCTGTCGACAGGCCTAGG -3′; downstream primer, 5′- TGTATCTTATCATGTCTGGATCCCC-3′. The XMRV SU fused to the human IgG Fc was generated using the two fragments as templates, and the upstream and downstream primer of the first and second fragment, respectively, and then the PCR product was cloned into the pCSI vector.

### Viruses and infection

The MoMLV retroviral pseudotypes encoding the alkaline phosphatase (AP) were produced by transfection of 293/GP-LAPSN cells with plasmid DNA encoding individual XMRV Env, CT truncations, or JSRV Env. The MoMLV retroviral pseudotypes encoding the green fluorescent protein (GFP) were generated by co-transfection of 293T cells with pCMV-gag-pol-MLV, pCMV-GFP-MLV (both vectors are kind gifts of François-Loïc Cosset) and plasmids encoding XMRV Env, XMRV Env CT truncations, Ebola GP (pCIneo-Ebola GP) [18], VSV-G (pMD.G), or MLV 10A1 Env (pCIneo-10A1) [18]. The HIV-1 lentiviral pseudotypes encoding AP were produced by co-transfecting 293T cells with pCMV-HIVΔ8.2, pHR'CMVAP [66] and plasmid DNA encoding individual Envs. All pseudotypes were harvested 48 and 72 h post-transfection and cell debris were removed by centrifugation at 2,500× g. MLV pseudovirions were purified by ultracentrifugation on a 20% sucrose cushion for 2 h at 185,000× g and 4°C, and Western blot was performed to examine SU incorporation using an anti-FLAG antibody. All viral infections were carried out in the presence of 5 µg/ml polybrene (Sigma) and viral titers were determined by AP staining or flow cytometry analysis to measure GFP^+^ cells 48–72 h post-infection. For infection in the presence of drugs or soluble XMRV SU or JSRV SU, cells were first pre-treated with the indicated concentrations of drugs at 37°C or the soluble proteins at 4°C for 1 h, and then incubated with retroviral pseudotypes for 6 h in the presence of drugs or fusion proteins before inactivation using citrate buffer (40 mM sodium citrate, 10 mM KCl, 135 mM NaCl, pH 3.15).

### Syncytium induction and cell-cell fusion assays

The syncytium induction assay was performed as described previously with some modifications [6], [19]. 293 cells were co-transfected with plasmids encoding XMRV Env or CT truncation mutants plus a GFP-encoding plasmid in order to monitor the transfection efficiency and syncytia formation. Syncytia formation was typically observed and photographed 24 h post-transfection. Where applicable, cells were treated for 5 minutes at 37°C with pre-warmed pH 5.0 buffer (phosphate-buffered saline (PBS), 10 mM MES, 10 m M HEPES) or 0.2–0.5 mM CPZ for 1 min and incubated in normal growth media at 37°C for 1 h.

The cell-cell fusion assay was performed as described previously [19], [45]. Briefly, effector 293T/GFP cells were transfected with plasmid DNA encoding XMRV Env or CT truncation mutants using Lipofectamine 2000 (Invitrogen). Twenty-four hours later, cells were washed with PBS and detached using PBS containing 5 mM EDTA. Target 293, 293/XPR1, CHO, or CHO/XPR1 cells were detached using PBS-5 mM EDTA and labeled with 3.5 µM CMTMR in serum-free media for 30 min at 37°C, washed, incubated for an additional 30 min at 37°C in fresh media and washed 3 times with media. Effector cells and target cells were co-cultured on 24-well plates for the indicated time periods. Cell-cell fusion was measured by flow cytometry using FACSCalibur (BD Bioscience, Missauga, Canada). The surface expression of XMRV Env in the 293T/GFP cells was measured by flow cytometry using the anti-FLAG antibody and anti-mouse IgG coupled to PE.

### Production of XMRV SU fusion protein and its binding to cells

Soluble XMRV SU and JSRV SU fusion proteins were produced as described previously [43]. 293T cells were transfected using the calcium-phosphate method with plasmids encoding the different SU. Twelve hours post-transfection, media were replaced with DMEM supplemented with 2% ultra-low IgG FBS (Invitrogen). The proteins in the media were purified using protein A beads (GE Healthcare, Uppsala, Sweden) and analyzed by SDS-PAGE and Sypro Ruby staining (Bio-Rad, Hercules, CA).

XMRV SU binding assays were performed as described previously [6], [43]. Cells were incubated with 2–10 µg of soluble XMRV SU-IgG fusion protein for 3–4 h on ice, washed 3 times with PBS-2% FBS and incubated with anti-human IgG coupled to FITC for detection. Fluorescence was measured by flow cytometry using FACSCalibur (BD Bioscience, Missauga, Canada).

### Immunostaining

CHO or CHO/XPR1 cells were fixed using 4% paraformaldehyde in PBS, permeabilized using 0.5% Triton X-100 and stained using anti-XPR1 and anti-rabbit IgG coupled to FITC. Before mounting the slides, cells were counterstained with the nuclear stain DAPI. Pictures were taken using a fluorescence microscope (Carl Zeiss, Goettingen, Germany) and images were processed using the ImageJ software (U.S., National Institutes of Health).

### Metabolic labeling

Metabolic labeling was performed as previously described [19], [45]. Briefly, 293T cells were transfected using the calcium-phosphate method with plasmid DNA encoding individual Env. Twenty-four hours later, cells were starved in cysteine and methionine-free DMEM for 30 minutes, pulse-labeled with 62.5 µCi ^35^S-cysteine and –methionine for 1 h at 37°C, washed with fresh media and chased for 4 h at 37°C in complete growth medium. Media were then collected and cells washed and lysed (50 mM Tris pH 8.0, 150 mM NaCl, 0.4 mM EDTA, 1% Triton X-100, 0.1% NP-40, 10 µg/ml aprotinin (Sigma), 10 µg/ml leupeptin (Sigma) and 1 mM phenylmethylsulfonyl fluoride (Sigma). The XMRV Env proteins in media and in cell lysates were immunoprecipitated using anti-FLAG beads and resolved by SDS-PAGE. Dried gels were autoradiographed and band intensities of XMRV SU in the cultured media were quantified using the Quantity One software (Bio-Rad, Hercules, CA).
